# Intensive Care Unit Mortality Trends during the First Two Years of the COVID-19 Pandemic in Greece: A Multi-Center Retrospective Study

**DOI:** 10.3390/v16040488

**Published:** 2024-03-22

**Authors:** Paraskevi C. Fragkou, Sotirios P. Karagiannis, Dimitra Dimopoulou, Sotiria Kefala, Fotini Fligou, Parisis Gallos, Edison Jahaj, Angeliki Bellou, Evangelia Koukaki, Eleni Magira, Philippos Orfanos, Georgios Papathanakos, Athanasios Papathanasiou, Emmanouil Pediaditis, Konstantinos Pontikis, Nikoletta Rovina, Katerina Vaporidi, Menelaos Xenikakis, Maria Theodorakopoulou, Anastasia Kotanidou

**Affiliations:** 1 First Department of Critical Care and Pulmonary Diseases, Evangelismos General Hospital of Athens, National and Kapodistrian University of Athens, 10676 Athens, Greece; sotiriskaragiann@gmail.com (S.P.K.); edison.jahaj@gmail.com (E.J.); elmagira@yahoo.com (E.M.); mariatheodor10@gmail.com (M.T.); akotanid@med.uoa.gr (A.K.); 2 Second Department of Pediatrics, “Aghia Sophia” Children’s Hospital, 11527 Athens, Greece; dimi_med@hotmail.com; 3 Division of Anesthesiology and Intensive Care Medicine, School of Medicine, University of Patras, 26504 Patras, Greece; swswkefala@gmail.com (S.K.); fflig@yahoo.com (F.F.); angelabelllou@gmail.com (A.B.); 4 Computational Biomedicine Laboratory, Department of Digital Systems, University of Piraeus, 18534 Piraeus, Greece; parisgallos@yahoo.com; 5 Intensive Care Unit, First Department of Respiratory Medicine, School of Medicine, National and Kapodistrian University of Athens, Sotiria Chest Hospital, 11527 Athens, Greece; e.koukaki@yahoo.gr (E.K.); kostis_pontikis@yahoo.gr (K.P.); nikrovina@uoa.gr (N.R.); 6 Department of Hygiene, Epidemiology and Medical Statistics, School of Medicine, National and Kapodistrian University of Athens, 11527 Athens, Greece; phorfanos@med.uoa.gr; 7 Department of Intensive Care Medicine, University Hospital of Ioannina, 45500 Ioannina, Greece; gppthan@icloud.com (G.P.); thanasis.papathanasiou@gmail.com (A.P.); mxenikakis@yahoo.gr (M.X.); 8 Department of Intensive Care Unit, University Hospital of Heraklion, School of Medicine, University of Crete, 70013 Heraklion, Greece; manospediaditis@gmail.com (E.P.); vaporidi@gmail.com (K.V.)

**Keywords:** COVID-19, SARS-CoV-2, ICU, mortality, Greece

## Abstract

Data on COVID-19 mortality among patients in intensive care units (ICUs) from Eastern and/or Southern European countries, including Greece, are limited. The purpose of this study was to evaluate the ICU mortality trends among critically ill COVID-19 patients during the first two years of the pandemic in Greece and to further investigate if certain patients’ clinical characteristics contributed to this outcome. We conducted a multi-center retrospective observational study among five large university hospitals in Greece, between February 2020 and January 2022. All adult critically ill patients with confirmed COVID-19 disease who required ICU admission for at least 24 h were eligible. In total, 1462 patients (66.35% males) were included in this study. The mean age of this cohort was 64.9 (±13.27) years old. The 28-day mortality rate was 35.99% (*n* = 528), while the overall in-hospital mortality was 50.96% (*n* = 745). Cox regression analysis demonstrated that older age (≥65 years old), a body mass index within the normal range, and a delay in ICU admission from symptom onset, as well as worse baseline clinical severity scores upon ICU admission, were associated with a greater risk of death. Mortality of critically ill COVID-19 patients was high during the first two years of the pandemic in Greece but comparable to other countries. Risk factors for death presented in this study are not different from those that have already been described for COVID-19 in other studies.

## 1. Introduction

The mortality rate of Coronavirus Disease 2019 (COVID-19) infection among hospitalized patients, and especially critically ill patients, was immense during the first two years of the pandemic. This phenomenon was attributed to several factors, including the pathophysiology of the disease itself, as well as the collapse of the healthcare systems around the globe, the cramped intensive care unit (ICU) capacities, and the limited available treatment options [1,2]. Older age, male gender, comorbidities such as immunosuppression, chronic lung diseases (including asthma and chronic obstructive pulmonary disease), cardiovascular diseases, diabetes, and chronic kidney or liver diseases, as well as a lower socioeconomic status, have been identified as risk factors associated with enhanced disease severity and, consequently, higher mortality rates [3]. 

Data (published mostly from developed high-income countries) have showed an improvement in the survival rates among COVID-19 patients during the subsequent waves, attributed to the changes in therapeutic strategies and management and the establishment of widespread vaccination programs around the globe [4,5,6,7]. However, this reduction in the mortality trends may not be representative for all countries. For instance, COVID-19-related mortality seems to be more pronounced in low- and middle-income countries compared to developed Western countries, as limited resources and the lack of healthcare infrastructures and capacities inevitably have a negative impact on optimized medical management and, eventually, survival [8,9,10].

Despite the unprecedented number of published articles during the pandemic, data on mortality trends among critically ill patients with COVID-19 remain sparse. To date, there are large COVID-19 registries evaluating the outcomes of critically ill patients with COVID-19 primarily in high-income countries with well-organized and resilient healthcare systems and facilities like the United States of America (USA), the United Kingdom (UK), and countries of Central and Northern Europe [11,12,13,14,15], but data on the mortality trends among ICU hospitalized patients with COVID-19 in other countries such as Eastern and/or Southern European countries, including Greece, are limited. Presenting ICU mortality data from countries which followed different public health policies and countries which had non-identical healthcare structures and responses to COVID-19 can help us to better understand the association between healthcare services and clinical outcomes. Moreover, understanding the variability in ICU surge response as well as recognizing the epidemiology and outcomes of ICU hospitalized patients are crucial in order to be better prepared for future pandemics. Hence, the purpose of this study is to evaluate the trends in ICU mortality among critically ill patients with confirmed COVID-19 during the first two years of the pandemic in Greece and to further investigate if patients’ clinical characteristics affect the final outcome.

## 2. Materials and Methods

### 2.1. Definitions

COVID-19 disease was confirmed through a positive RT-PCR on either nasopharyngeal or bronchial aspiration sample or other respiratory sample. 

During the period of this study, there were four different pandemic waves: the 1st wave: February 2020–July 2020, the 2nd wave: August 2020–December 2020, the 3rd wave: January 2021–August 2021, and the 4th wave: September 2021–January 2022. 

The following comorbidities were accounted: asthma, chronic pulmonary obstructive disease long-term oxygen therapy, coronary artery disease, other cardiovascular diseases (e.g., cerebrovascular accident, transient ischemic attack, peripheral vascular disease), dyslipidemia, diabetes mellitus (both insulin and non-insulin dependent), hypertension, hematological or solid organ malignancy (active or within the last 5 years), recent (within 6 months) chemotherapy, recent (within 6 months) or current administration of immunosuppressive drugs, and chronic kidney disease (eGRF ≤ 89 mL/min/1.73 m^2^). 

Overall mortality was defined as in-hospital mortality irrespective of whether death happened in the ICU or on the ward after discharge from the ICU, while 28-day mortality was in-hospital mortality within the first 28 days of ICU admission irrespective of whether death happened in ICU or on the ward after discharge from the ICU.

### 2.2. Patient Selection and Data Collection

We conducted a multi-center retrospective observational study of adult critically ill patients with confirmed COVID-19 disease admitted in the ICU in one of the five tertiary hospitals in Greece, between February 2020 and January 2022. Evangelismos General Hospital of Athens (EGH), Sotiria Chest Hospital (SCH), University Hospital of Heraklion (UHH), University Hospital of Ioannina (UHI), and University Hospital of Patras (UHP) were the hospitals involved in this study. All five institutions are large university hospitals that served as COVID-19 referral centers during the pandemic. Patients’ inclusion criteria were the following: (1) age ≥ 18 years and (2) confirmed SARS-CoV-2 infection by Reverse Transcription Polymerase Chain Reaction (RT-PCR) requiring admission in ICU for at least 24 h. Patients not meeting all the aforementioned inclusion criteria and/or with multiple missing data were excluded from this study. 

Data were retrieved by the hospitals’ medical records. Before the study began, preliminary data collection was conducted by the investigators of each site, in order to assess the data compatibility and to ensure that a homogeneous data collection process would be followed among all participating institutions. All data were extracted to CSV files, excluding the personal details of the patients (patient identification numbers were used for data-tracking purposes). Collected data included: age, gender, date of symptom onset, date of hospital admission, date of ICU admission, vaccination status, need for intubation and mechanical ventilation, specific laboratory results, duration of hospitalization and length of ICU stay, 28-day mortality, overall in hospital mortality, Acute Physiology and Chronic Health Evaluation (APACHE) II score, body mass index (BMI), comorbidities [such as: smoking status (active smokers/ex-smokers and non-smokers), diabetes mellitus, hypertension, cardiovascular disease, chronic obstructive pulmonary disease (COPD), etc.], and complications related to COVID-19.

This study was conducted and reported according to the Strengthening the Reporting of Observational Studies in Epidemiology (STROBE) statement [16].

### 2.3. Ethical Statement

This study was approved by the Research Ethics Committee of the participating institutions (approval protocol numbers: EGH: 26/20-1-2022; SCH: 23464/8/9/20; UHH: 7188/07-07-2021; UHI: 8/30-03-2022(θ.20); UHP: 119/10.03.2022). Patients’ data were extracted, analyzed, and handled under strict anonymity in agreement with the Helsinki Declaration. Since this was a retrospective study, the need for signed informed consent was waived by the Research Ethics Committees of all participating institutions.

### 2.4. Statistical Analysis

Age (<65 years old, ≥65 years old, N%), sex (male, female, N%), wave of admission (1st wave, 2nd wave, 3rd wave, 4th wave, N%), total number of comorbidities (<2, ≥2, N%), smoking status (never, former, current, N%), BMI (normal, overweight, obese, N%), and use of steroids (dexamethasone, no dexamethasone, N%) were analyzed as categorical variables. Days from symptom onset to ICU admission and APACHE II score were analyzed as continuous variables. 

Descriptive statistics were calculated to examine the distribution of the variables. Continuous variables were tested using a Mann–Whitney U test in order to examine their relationship with mortality. For dichotomous variables, a chi-squared test was used. For mortality, we defined a priori the following variables as potential confounders: age, sex, body mass index, and wave of admission. We examined the effects of variables on mortality by univariate analysis, and then the effect of several variables was tested through both univariate Cox regression models and multivariate models, adjusted for potential confounders. Schoenfeld residuals were used to test the assumption of proportional hazards and found that it was not violated. Patients with at least 1 missing value were excluded from the Cox regression analysis.

## 3. Results

### 3.1. Patient Characteristics and Demographics

The current cohort was comprised of 1462 ICU patients with a mean (±SD) age of 64.9 (±13.27) years old. Among them, 970 were males (66.35%). The largest portion of the patients (725, 49.35%) was admitted to the ICU during the third wave of the pandemic. Half of the patients were admitted to the ICU by the 10th day of symptom onset [Interquartile range (IQR): 7, 14]. The median duration of hospitalization in the ICU was 14 days (IQR: 7, 28). As far as the clinical severity scores are concerned, median (IQR) SOFA and APACHE II scores were 6 (IQR:3, 9) and 14 (IQR:10, 20), respectively. Among all patients, 743 (51.92%) had at least two comorbidities (Table 1). 

Patients were divided according to overall mortality into the “survivors” and “non-survivors”. “Non-survivors” were significantly older than “survivors” with a median age (IQR) of 71 (64–78) years old and 61 (52–69) years old (*p* < 0.001), respectively (Table 1). Male patients accounted for 66.34% (*n* = 475) of the “survivors” and 66.44% (*n* = 495) of the “non-survivors” (*p* = 0.967). More than 70% of the patients were admitted during the second and third waves. In contrast to the first three waves, non-survivors (33.42%) were twice as many as survivors (17.15%) during the fourth wave. Half of the survivors were admitted to the ICU by the 10th day (IQR: 7–13) of symptoms onset while half of the non-survivors were admitted by the 11th day (IQR: 7, 15.5) of symptoms onset (*p* = 0.017). Additionally, “non-survivors” had significantly more comorbidities, they were more severely ill upon admission (based on the SOFA and APACHEII scores), and developed more complications compared to the “survivors”. Acute kidney injury, need for continuous venovenous hemodiafiltration (CVVHDF), and bacteremia were observed more frequently in the “non-survivors” group (Table 1).

### 3.2. 28-Day Mortality

**Figure 1:**
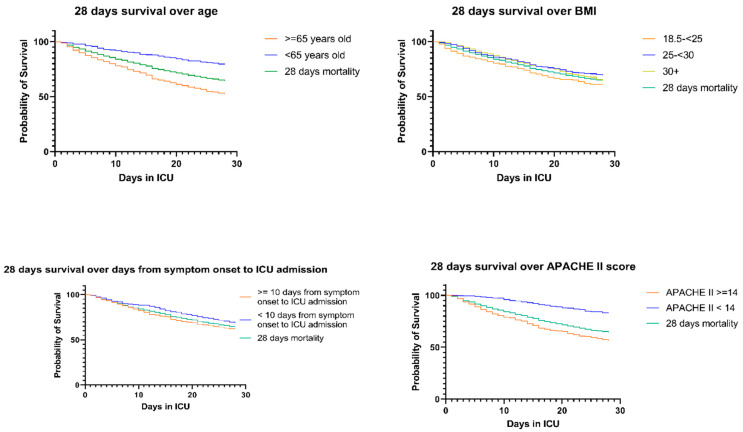
Kaplan–Meier curves for 28-day survival over age, BMI, days from symptom onset, and APACHE II score.

In the current study, the 28-day mortality rate was 35.99% (*n* = 528). In the multivariate Cox regression model, age over 65 years significantly increased the odds of death by 226% (*p* < 0.001) (Table 2, Figure 1). There was no significant association between gender and 28-day mortality. Patients with BMI between 25 and 30 kg/m^2^, as well as patients with BMI over 30 kg/m^2^, were found to have significantly less risk for death—HR 0.71 and 0.76, respectively—when compared to patients with normal BMI. The number of days from symptom onset to ICU admission increased the risk of death by 2% (*p* < 0.001). Higher APACHE II score upon admission was associated with an HR of 1.03 (*p* < 0.001). Active smoking status increased the risk by 36% (*p* = 0.058). The number of total comorbidities and the use of dexamethasone were not found to significantly alter the risk of death in the first 28 days of ICU hospitalization. Multivariate logistic regression of overall morbidity, 28-days morbidity and overall ICU-morbidity are presented in Tables S1–S3. Figures S1 and S2 present Kaplan-Meier curves for overall and 28 days mortality, respectively.

### 3.3. Overall In-Hospital Mortality

In this cohort, the overall in-hospital mortality was 50.96% (*n* = 745). The Cox regression model for overall mortality (Table 3) among COVID-19 ICU patients demonstrated that an age of ≥65 years old increased the risk of death by 80% (*p* < 0.001). Additionally, subjects admitted to the ICU during the fourth wave of the pandemic had a high HR (1.91, *p* = 0.068) for death, without reaching statistical significance. Additionally, each day of ICU admission delay increased the risk of death by 2% (*p* < 0.001). Regarding the clinical severity of the patients upon admission, we found an associated HR of 1.03 (*p* < 0.001) for APACHEII.

## 4. Discussion

In this multi-center retrospective cohort, 1462 patients with laboratory-confirmed COVID-19 requiring ICU admission from five different large university hospitals in Greece were included. The overall in-hospital and the 28-day mortality rates were 50.96%, and 35.99%, respectively. Our data demonstrated that older age, BMI within the normal range, admission to the ICU later in the course of the disease, and worse clinical condition (as depicted by higher APACHEII and SOFA scores) at baseline were statistically significant risk factors for death in hospital, including the first 28 days of ICU stay. 

Most of the patients of our cohort were men (66.4%) with a median (IQR) age of 67 (57–74) years old and had at least one major comorbidity, as defined by our protocol (see Section 2.1). These findings are in line with previously published data showing that older age and distinct comorbidities (such as dyslipidemia, diabetes mellitus, chronic obstructive pulmonary disease, hypertension, or heart disease) are associated with a poor outcome in critically ill COVID-19 patients [3,17]. 

Besides age and comorbidities, our study showed a statistically significant correlation between overall mortality, 28-day mortality and overall ICU mortality, and the days from symptom onset to ICU admission. Patients who were admitted later to the ICU were at a greater risk for death. These results may underly the importance of the prompt implementation of intensive care in patients with COVID-19 requiring intubation in reducing mortality. In contrast to our results, the study from Grasselli et al. examined the mortality among patients with COVID-19 requiring admission to the ICU in Lombardi, Italy, and did not find a significant correlation between the overall mortality and the days from symptoms’ onset until ICU admission [3]. Although the median time elapsed between the initiation of symptoms and ICU admission was 10 days, as in our study, it should be considered that this study was conducted during the first two months of the pandemic (February 2020 to April 2020). Understandably, many factors could have contributed to the observed difference in the outcomes, including the virulence of the circulating variant or the different care that was provided before the admission to the ICU between the two countries.

In contrast to previously published data [3,17,18,19], we gathered and analyzed data from March 2020 to January 2022; hence, we compared the outcomes of interest between the different waves of the pandemic. Our study demonstrated that overall mortality and 28-day mortality were higher during the fourth wave (August 2021–January 2022), when the Delta variant was dominated in Greece. The analysis revealed that patients during this time period were significantly older, were admitted later to the ICU, and they had higher SOFA and APACHE II scores. Previous studies also reported a higher mortality rate in parallel with the surge of the Delta strain, as a result of the older age of the patients included in these cohorts and the delayed ICU admission [18,19]. It is worth noting that the Delta variant causes more severe disease and is characterized by higher transmissibility compared to the previous variants of concern [20,21].

Unambiguously, there is some geographical variation in COVID-19-related mortality among countries due to the difference in healthcare resources and infrastructures; however, it has been suggested that the overall mortality has plateaued or even reduced since May 2020 [13]. In our study, we observed a higher mortality during the Delta variant’s dominance in Greece, compared to previous waves of the pandemic. Other studies have also reported similar mortality rates (30–40%) in the majority of geographical regions, apart from Middle Eastern countries [13]. This phenomenon may be partially attributed to the differences in healthcare systems, which in turn reflect the variations in critical care services among different countries. Importantly, a study by Tabatabai et al., which collected data from critically ill COVID-19 patients who were admitted to the ICU between January 2020 and March 2022 across 57 states of the USA, showed that mortality during the Omicron dominance period was lower than mortality during previous COVID-19 waves [22]. To our knowledge, there are no published data about the mortality of critically ill patients after March 2022. Hence, evidence on ICU mortality among the different waves remains conflicting, and more studies are needed to shed light on the differences in mortality among different waves and countries. 

Vaccinated patients made up only a very small fraction of the patients requiring ICU admission in this cohort. We identified that patients who were vaccinated had significantly more comorbidities, and they were more severely ill upon admission (scoring higher both at APACHE II and SOFA scores), compared to the non-vaccinated subjects (Supplementary Tables S4–S6).

Our study has several strengths. First, this study was carried out in five university hospitals (which were referral centers for COVID-19) in different regions of Greece. This ensured that all patients received an equal level of healthcare services. Moreover, the large number of patients included in this study allowed for further subgroup analyses that helped us identify potential confounders and test for associations of several variables with mortality. Additionally, the dominant SARS-CoV-2 variants of concern changed during the study period. Besides the circulating strains, treatment options evolved and drastically changed within these 2 years. This allowed us to compare not only the outcomes of interest according to the different circulating variants but also according to the different therapeutic interventions that were recommended during this period, leading to a more holistic approach to our primary outcome.

However, our study has some limitations. First of all, it is a retrospective study. Although we have recorded many different variables—laboratory data, clinical severity scores, COVID-19- and ICU-related complications, and data regarding the COVID-19 treatment plan—we cannot exclude the possibility of unmeasured confounders that may affect the results. Secondly, the data were extracted from an electronic real-life database. The physical strain of the healthcare professionals and the medical system as a whole during the first waves of the pandemic inevitably led to omissions in the electronic records and, obviously, to missing data. Thirdly, the medical records of the patients are available only for the time frame of their stay in each hospital. There are no data available to us regarding the clinical outcomes of patients after their discharge. Finally, most patients with COVID-19 requiring intubation were treated in university hospitals. Consequently, this could have introduced selection and indication bias, and it could also have affected the generalizability of this study.

## 5. Conclusions

In conclusion, in this study we demonstrated that mortality rates among COVID-19 patients requiring ICU admission in Greece during the first two years of the pandemic were similar to those that have been previously reported in other countries. Additionally, we found that clinical characteristics such as older age, increased body mass index, and delay in ICU admission from symptom onset, as well as worse baseline clinical severity scores, were significantly associated with a higher risk of death, as has been described in other COVID-19 cohorts. Hence, our data denote that COVID-19 mortality among patients requiring ICU admission was comparable among countries with divergent levels of healthcare services that implemented different public health measures and policies.

## Figures and Tables

**Table 1:** Patient characteristic and demographics by overall outcome.

	Survivors N = 717	Non-Survivors N = 745	*p*-Value
Demographics			
Age, median (IQR) [years]	61 (52–69)	71 (64–78)	*<0.001*
Sex, *n* (%) (1 missing)	Male: 475 (66.34%)	Male: 495 (66.44%)	0.967
Wave, *n* (%) (0 missing)	1st: 25 (3.49%) 2nd: 170 (23.71%) 3rd: 399 (55.65%) 4th: 123 (17.15%)	1st: 18 (2.42%) 2nd: 154 (20.67%) 3rd: 324 (43.49%) 4th: 249 (33.42%)	*<0.001*
Comorbidities			
BMI (kg/m^2^) *n* (%) (0 missing)	18.5–24: 304 (42.40%) 25–29: 197 (27.48%) 30–34: 109 (15.20%) 35–39: 64 (8.93%) ≥40: 43 (6.00%)	18.8- < 25: 298 (40.00%) 25- < 30: 203 (27.25%) 30- < 35: 143 (19.19%) 35- < 40: 47 (6.31%) 40+: 54 (7.25%)	0.090
Smoking Status, *n* (%) (129 missing)	Never: 458 (66.38%) Former: 159 (23.04%) Current: 73 (10.58%)	Never: 401 (61.50%) Former: 164 (25.15%) Current: 87 (13.34%)	0.135
Hypothyroidism, *n* (%) (53 missing)	106 (14.91%)	65 (9.19%)	0.001
Immunosuppression, *n* (%) (50 missing)	25 (3.52%)	49 (6.90%)	0.004
Diabetes Mellitus, *n* (%) (47 missing)	159 (22.33%)	210 (29.49%)	*0.002*
Coronary Artery Disease, *n* (%) (47 missing)	61 (8.57%)	144 (20.22%)	*<0.001*
Hypertension, *n* (%) (47 missing)	332 (46.63%)	405 (56.88%)	*<0.001*
Cancer/Hematologic Malignancy, *n* (%) (47 missing)	74 (10.39%)	138 (19.38%)	*<0.001*
Chronic Obstructive Pulmonary Disease, *n* (%) (47 missing)	34 (4.77%)	99 (13.90%)	*<0.001*
Chronic Kidney Disease on Renal Replacement Therapy, *n* (%) (110 missing)	19 (2.76%)	68 (10.10%)	*<0.001*
Disease status			
Days from symptom onset to hospital admission, median (IQR) (51 missing)	7 (5–9)	5 (3–8)	*<0.001*
Days from symptom onset to ICU, median (IQR) (36 missing)	10 (7–13)	11 (7–15.5)	*0.017*
SOFA at ICU admission, median (IQR) (269 missing)	4 (2–7)	8 (5–9)	*<0.001*
APACHEII at ICU admission, median (IQR) (249 missing)	11 (8–16)	18 (13–24)	*<0.001*
Complications during ICU stay			
Intubation, *n*(%) (11 missing)	446 (62.20%)	734 (98,79%)	*<0.001*
Acute Kidney Injury, *n* (%) (52 missing)	Stage 1: 75 (10.58%) Stage 2: 23 (3.24%) Stage 3: 57 (8.04%)	Stage 1: 97 (13.66%) Stage 2: 98 (13.80%) Stage 3: 281 (39.58%)	*<0.001*
Continuous Venovenous Hemodiafiltration, *n* (%) (52 missing)	60 (8.42%)	276 (39.09%)	*<0.001*
Bacteremia, *n* (%) (9 missing)	206 (28.73%)	456 (61.21%)	*<0.001*

BMI: body mass index; IQR: interquartile range; ICU: intensive care unit; CRP: C-reactive protein; WBCs: white blood cells; APACHEII: Acute Physiology and Chronic Health Evaluation II; SOFA: Sequential Organ Failure Assessment.

**Table 2:** Univariate and multivariate Cox regression of 28-day mortality.

	Univariate Analysis		Multivariate Analysis	
Variables	Hazard Ratio (95% CI)	*p*-Value	Hazard Ratio (95% CI)	*p*-Value
Age				
≥65 years old	2.74 (2.54, 3.34)	<0.001	2.26 (1.77, 2.89)	<0.001
Sex				
Female	1.06 (0.89, 1.27)	0.514	0.83 (0.66, 1.05)	0.123
Wave of admission				
2nd wave	0.99 (0.56, 1.77)	0.994	1.01 (0.45, 2.29)	0.973
3rd wave	0.90 (0.51, 1.58)	0.723	1.01 (0.46, 2.23)	0.979
4th wave	1.92 (1.09, 3.36)	0.023	1.64 (0.74, 3.60)	0.220
BMI				
25–29	0.72 (0.58, 0.89)	0.004	0.71 (0.54, 0.93)	0.012
≥30+	0.84 (0.68, 1.02)	0.084	0.76 (0.58, 0.98)	0.037
Total number of comorbidities				
≥2	1.70 (1.41, 2.04)	<0.001	1.14 (0.91, 1.42)	0.255
Smoking status				
Former	1.03 (0.82, 1.29)	0.803	0.98 (0.76, 1.26)	0.880
Current	1.36 (1.04, 1.77)	0.026	1.36 (0.99, 1.87)	0.058
Days from symptom onset to ICU admission	1.03 (1.02, 1.04)	<0.001	1.02 (1.01, 10.4)	<0.001
APACHE II score upon ICU admission	1.02 (1.01, 1.03)	<0.001	1.04 (1.03, 1.04)	<0.001
Steroids				
Dexamethasone	0.81 (0.66, 0.99)	0.040	0.92 (0.70, 1.21)	0.549

**Table 3:** Univariate and multivariate Cox regression of overall in-hospital mortality.

	Univariate Analysis		Multivariate Analysis	
Variables	Hazard Ratio (95% CI)	*p*-Value	Hazard Ratio (95% CI)	*p*-Value
Age				
≥65 years old	2.16 (1.83, 2.54)	<0.001	1.79 (1.47, 2.20)	<0.001
Sex				
Female	1.18 (1.01, 1.37)	0.037	0.98 (0.80, 1.19)	0.835
Wave of admission				
2nd wave	1.61 (0.99, 2.63)	0.056	1.57 (0.77, 3.22)	0.216
3rd wave	1.36 (0.85, 2.19)	0.206	1.45 (0.72, 2.89)	0.296
4th wave	2.14 (1.32, 3.46)	0.002	1.91 (0.96, 3.82)	0.068
BMI				
25–29	0.94 (0.79, 1.23)	0.508	0.89 (0.71, 1.12)	0.322
≥30	1.05 (0.88, 1.24)	0.582	0.96 (0.77, 1.20)	0.740
Total number of comorbidities				
≥2	1.41 (1.21, 1.64)	<0.001	1.08 (0.89, 1.29)	0.434
Smoking status				
Former	1.05 (0.88, 1.27)	0.538	1.00 (0.81, 1.24)	0.970
Current	1.20 (0.95, 1.52)	0.120	1.17 (0.90, 1.54)	0.243
Days from symptom onset to ICU admission	1.01 (1.01, 1.02)	<0.001	1.02 (1.01, 1.03)	<0.001
APACHE II score upon ICU admission	1.04 (1.03, 1.05)	<0.001	1.03 (1.02, 1.04)	<0.001
Steroids				
Dexamethasone	0.93 (0.79, 1.11)	0.457	1.06 (0.85, 1.33)	0.615

## Data Availability

The data underlying this article will be shared upon reasonable request to the corresponding author.

## Supplementary Materials

The following supporting information can be downloaded at: https://www.mdpi.com/article/10.3390/v16040488/s1, Table S1. Multivariate Logistic regression of overall morbidity, Table S2. Multivariate Logistic regression of 28-days morbidity, Table S3. Multivariate Logistic regression of overall ICU morbidity, Table S4. Chi-square test for vaccination status and total number of comorbidities, Table S5. Two-sample Wilcoxon rank-sum test for APACHE II score between vaccinated and non-vaccinated patients, Table S6. Two-sample Wilcoxon rank-sum test for SOFA score between vaccinated and non-vaccinated patients, Figure S1. Kaplan-Meier curves for overall mortality over different variables, Figure S2. Kaplan-Meier curves for 28 days mortality over different variables.
