# Supplementary material for: Intensive Care Unit Mortality Trends during the First Two Years of the COVID-19 Pandemic in Greece: A Multi-Center Retrospective Study
Source: Viruses. 2024 Mar 22;16(4):488. doi: 10.3390/v16040488 (PMC11054592; doi:10.3390/v16040488)

Supplementary Materials

Suppl Table S1. Multivariate Logistic regression of overall morbidity.

	Coef.	St.Err.	t-value	p-value	[95% Conf Interval]		Sig
Age	1	.	.	.	.	.	
≥65	2.995	0.478	6.87	0	2.191	4.095	***
Sex	1	.	.	.	.	.	
Female	0.712	0.117	-2.07	0.038	0.516	0.982	**
Wave	1	.	.	.	.	.	
2nd wave	4.138	2.184	2.69	0.007	1.471	11.643	***
3rd wave	2.966	1.502	2.15	0.032	1.099	8.001	**
4th wave	5.285	2.724	3.23	0.001	1.925	14.512	***
Days from symptom onset to ICU admission	1.053	0.013	4.10	0	1.027	1.079	***
APACHE II	1.09	0.016	5.97	0	1.059	1.121	***
SOFA	1.248	0.038	7.31	0	1.176	1.325	***
BMI	1	.	.	.	.	.	
25-29	1.455	0.281	1.94	0.053	0.996	2.125	*
30-34	2.003	0.434	3.20	0.001	1.309	3.063	***
35-39	.804	0.245	-0.72	0.473	0.443	1.46	
≥40	2.144	0.693	2.36	0.018	1.137	4.041	**
Total comorbidities	1	.	.	.	.	.	
≥2	1.342	0.206	1.92	0.055	0.993	1.814	*
Smoking status	1	.	.	.	.	.	
Current	1.207	0.289	0.79	0.431	0.756	1.93	
Ex-smoker	1.043	0.186	0.23	0.814	0.735	1.479	
Steroids administration	1	.	.	.	.	.	
Dexamethasone	0.675	0.134	-1.98	0.048	0.458	0.997	**
Vaccination status	1	.	.	.	.	.	
Yes	1.184	0.386	0.52	0.604	0.625	2.243	

*** $p < 0.01$, ** $p < 0.05$, * $p < 0.1$

BMI: Body mass index; ICU: Intensive Care Unit; APACHEII: Acute Physiology And Chronic Health Evaluation II; SOFA: Sequential Organ Failure Assessment.

Suppl. Table S2. Multivariate Logistic regression of 28-days morbidity.

	Coef.	St.Err.	t-value	p value	[95% Conf Interval]		Sig
Age	1	.	.	.	.	.	
≥65	2.459	0.413	5.36	0	1.77	3.417	***
Sex	1	.	.	.	.	.	
Female	0.896	0.146	-0.67	0.5	0.651	1.233	
Wave	1	.	.	.	.	.	
2nd wave	2.022	1.076	1.32	0.186	0.712	5.74	
3rd wave	1.3	0.664	0.51	0.608	0.478	3.539	
4th wave	2.232	1.146	1.56	0.118	0.816	6.108	
Days from symptom onset to	1.054	0.012	4.51	0	1.03	1.078	***

ICU admission							
APACHE II	1.037	0.013	2.90	0.004	1.012	1.063	***
SOFA	1.276	0.04	7.81	0	1.2	1.356	***
BMI	1	.	.	.	.	.	
25-29	0.821	0.16	-1.01	0.314	0.56	1.204	
30-34	1.286	0.273	1.19	0.236	0.848	1.949	
35-39	0.535	0.172	-1.95	0.052	0.285	1.004	*
≥40	1.201	0.389	0.56	0.572	0.636	2.265	
Total comorbidities	1	.	.	.	.	.	
>=2	1.128	0.176	0.77	0.442	0.83	1.532	
Smoking status	1	.	.	.	.	.	
Current	1.358	0.321	1.29	0.196	0.855	2.157	
Ex-smoker	1.015	0.181	0.08	0.935	0.715	1.441	
Steroids administration	1	.	.	.	.	.	
Dexamethasone	0.993	0.195	-0.04	0.971	0.676	1.459	
Vaccination status	1	.	.	.	.	.	
Yes	1.415	0.415	1.18	0.237	0.796	2.514	

*** $p < 0.01$, ** $p < 0.05$, * $p < 0.1$

BMI: Body mass index; ICU: Intensive Care Unit; APACHEII: Acute Physiology And Chronic Health Evaluation II; SOFA: Sequential Organ Failure Assessment.

Suppl. Table S3. Multivariate Logistic regression of overall ICU morbidity.

	Coef.	St.Err.	t-value	p-value	[95% Conf Interval]		Sig
Age	1	.	.	.	.	.	
≥65	2.925	0.464	6.76	0	2.143	3.992	***
Sex	1	.	.	.	.	.	
Female	0.702	0.114	-2.18	0.029	0.51	0.964	**
Wave	1	.	.	.	.	.	
2nd wave	4.413	2.335	2.81	0.005	1.564	12.449	***
3rd wave	3.114	1.585	2.23	0.026	1.148	8.447	**
4th wave	5.866	3.036	3.42	0.001	2.127	16.178	***
Days from symptom onset to ICU admission	1.057	0.013	4.48	0	1.032	1.083	***
APACHE II	1.074	0.015	5.24	0	1.046	1.104	***
SOFA	1.246	0.037	7.34	0	1.175	1.321	***
BMI	1	.	.	.	.	.	
25-29	1.148	0.218	0.73	0.468	0.791	1.667	
30-34	1.719	0.365	2.55	0.011	1.134	2.606	**
35-39	.676	0.203	-1.30	0.193	0.375	1.219	
≥40	1.619	0.518	1.51	0.132	0.865	3.03	
Total comorbidities	1	.	.	.	.	.	
≥2	1.215	0.185	1.28	.2	0.902	1.638	
Smoking status	1	.	.	.	.	.	
Current	1.315	0.311	1.16	0.246	0.827	2.09	
Ex-smoker	0.922	0.163	-0.46	0.646	0.653	1.303	
Steroids administration	1	.	.	.	.	.	
Dexamethasone	0.681	0.132	-1.98	0.048	0.465	0.997	**
Vaccination status	1	.	.	.	.	.	

Yes	1.284	0.413	0.78	0.437	0.684	2.411

*** $p < 0.01$, ** $p < 0.05$, * $p < 0.1$

BMI: Body mass index; ICU: Intensive Care Unit; APACHEII: Acute Physiology And Chronic Health Evaluation II; SOFA: Sequential Organ Failure Assessment.

Suppl. Table S4. Chi-square test for vaccination status and total number of comorbidities

Vaccination status	Total comorbidities		
	<2	≥2	Total
No	647	665	1312
Yes	40	77	117
Total	687	742	1429
Pearson Chi^2 = 9.85, p value = 0.0017			

Suppl. Table S5. Two-sample Wilcoxon rank-sum test for APACHE II score between vaccinated and non-vaccinated patients.

```
         vax |      obs    rank sum    expected
-------------+---------------------------------
          No |     1143    689228.5    702373.5
         Yes |       85     65377.5     52232.5
-------------+---------------------------------
    combined |     1228      754606      754606

unadjusted variance   9950291.25
adjustment for ties    -22594.87
                      ----------
adjusted variance     9927696.38

Ho: apache(vax==No) = apache(vax==Yes)
             z =  -4.172
    Prob > |z| =   0.0000
```

Suppl. Table S6. Two-sample Wilcoxon rank-sum test for SOFA score between vaccinated and non-vaccinated patients.

```
         vax |      obs    rank sum    expected
-------------+---------------------------------
          No |     1123    668053.5    678853.5
         Yes |       85     62182.5     51382.5
-------------+---------------------------------
    combined |     1208      730236      730236

unadjusted variance   9617091.25
adjustment for ties   -183846.83
                      ----------
adjusted variance     9433244.42

Ho: sofa(vax==No) = sofa(vax==Yes)
             z =  -3.516
    Prob > |z| =   0.0004
```

Suppl. Figure S1. Kaplan-Meier curves for overall mortality over different variables

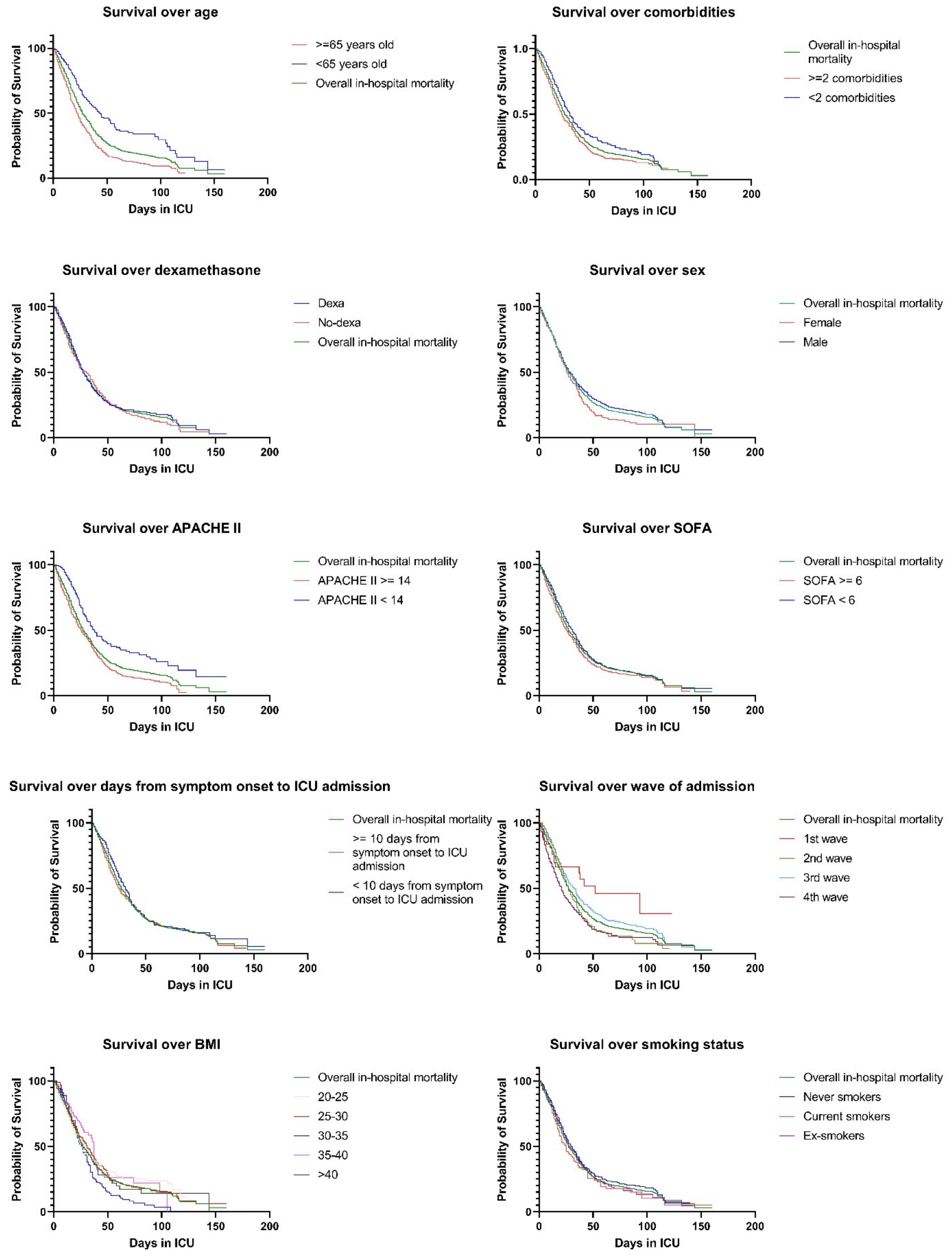

Suppl Figure S2. Kaplan-Meier curves for 28 days mortality over different variables

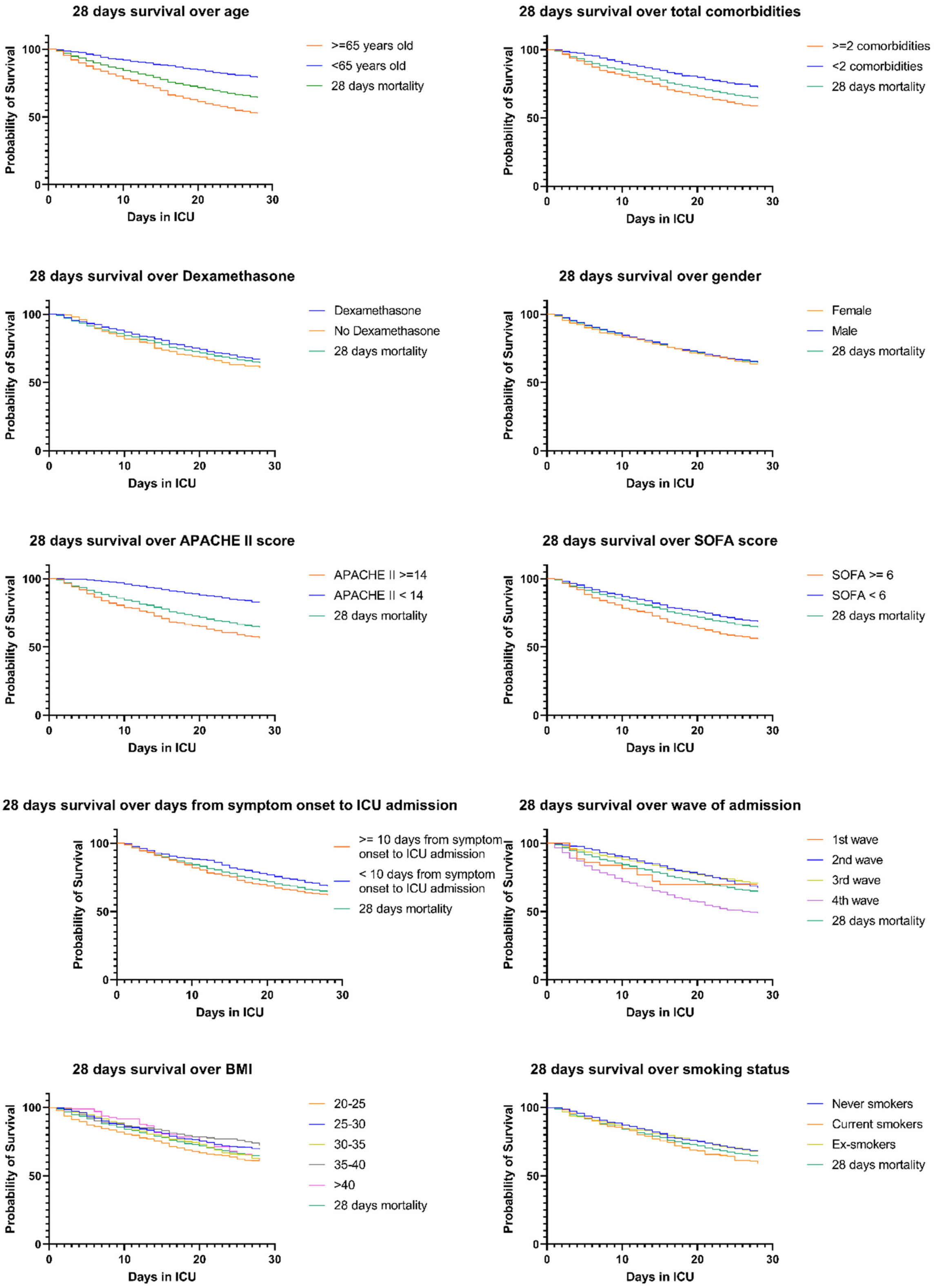

Supplement: Supplementary file 1 [file viruses-16-00488-s001.zip › viruses-2897034-supplementary.pdf]
